# Copper Organometallic Iodide Arrays for Efficient X-ray Imaging Scintillators

**DOI:** 10.1021/acscentsci.2c01495

**Published:** 2023-03-10

**Authors:** Hong Wang, Jian-Xin Wang, Xin Song, Tengyue He, Yang Zhou, Osama Shekhah, Luis Gutiérrez-Arzaluz, Mehmet Bayindir, Mohamed Eddaoudi, Osman M. Bakr, Omar F. Mohammed

**Affiliations:** † Advanced Membranes and Porous Materials Center, Division of Physical Science and Engineering, King Abdullah University of Science and Technology, Thuwal 23955-6900, Kingdom of Saudi Arabia; ‡ KAUST Catalysis Center, Division of Physical Sciences and Engineering, King Abdullah University of Science and Technology, Thuwal 23955-6900, Kingdom of Saudi Arabia; § Center for Hybrid Nanostructures, University of Hamburg, 22761 Hamburg, Germany

## Abstract

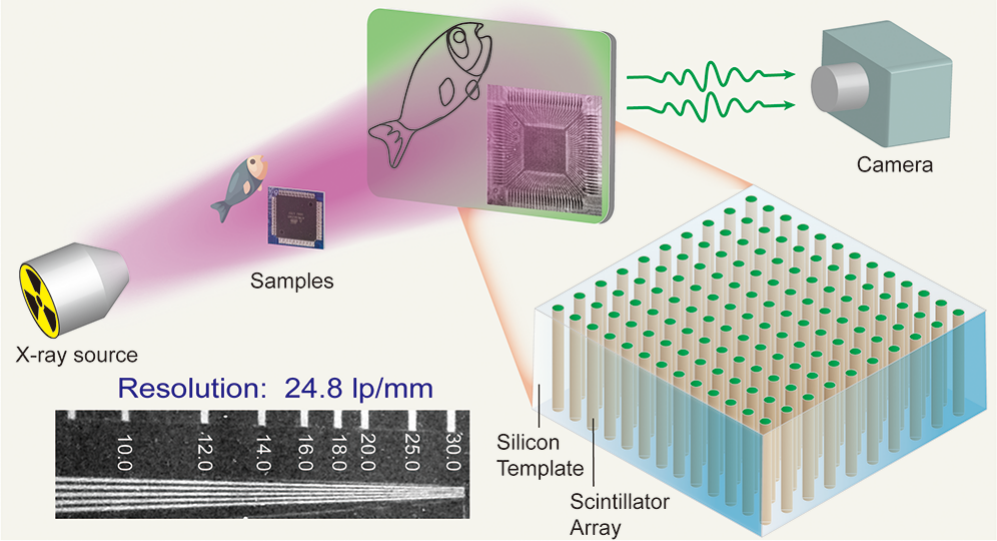

Lead-free organic metal halide scintillators with low-dimensional electronic structures have demonstrated great potential in X-ray detection and imaging due to their excellent optoelectronic properties. Herein, the zero-dimensional organic copper halide (18-crown-6)_2_Na_2_(H_2_O)_3_Cu_4_I_6_ (CNCI) which exhibits negligible self-absorption and near-unity green-light emission was successfully deployed into X-ray imaging scintillators with outstanding X-ray sensitivity and imaging resolution. In particular, we fabricated a CNCI/polymer composite scintillator with an ultrahigh light yield of ∼109,000 photons/MeV, representing one of the highest values reported so far for scintillation materials. In addition, an ultralow detection limit of 59.4 nGy/s was achieved, which is approximately 92 times lower than the dosage for a standard medical examination. Moreover, the spatial imaging resolution of the CNCI scintillator was further improved by using a silicon template due to the wave-guiding of light through CNCI-filled pores. The pixelated CNCI-silicon array scintillation screen displays an impressive spatial resolution of 24.8 line pairs per millimeter (lp/mm) compared to the resolution of 16.3 lp/mm for CNCI-polymer film screens, representing the highest resolutions reported so far for organometallic-based X-ray imaging screens. This design represents a new approach to fabricating high-performance X-ray imaging scintillators based on organic metal halides for applications in medical radiography and security screening.

## Abstract 2

We report a high-performance X-ray imaging scintillator based on a zero-dimensional organic copper halide-silicon arrays for applications in medical radiography and security screening.

X-ray imaging scintillators have been extensively used in the fields of medical diagnosis, defense security, and nondestructive inspection.^1−6^ In a typical X-ray scintillation process, X-ray absorption, photogeneration of charge carriers, and radiative carrier recombination are the key steps that lead to radioluminescence.^1^ Therefore, a high-performance scintillator material needs to meet at least two basic requirements: strong X-ray absorption and high photoluminescence quantum yield (PLQY). To date, many all-inorganic lead halide perovskites materials, such as CsPbX_3_ (X = I^–^, Br^–^, Cl^–^, or mixed halides) and related low-dimensional structures, have been developed as high-performance scintillators due to their high X-ray absorption, low detection limit, and good spatial imaging resolution.^7−11^ However, the toxicity of lead in these halide perovskites and the perovskites’ notorious instability under humid conditions have greatly limited the materials’ further development and commercialization.^12,13^ Therefore, exploring lead-free materials with high stability, low toxicity, low cost, high X-ray absorption cross-section, and high light yield for high-performance X-ray imaging scintillators is urgently needed.

To address these shortcomings, various eco-friendly lead-free metal halide scintillators, such as Cu(I)^−^, Ag(I) ^–^, Mn(II)^−^, Sb(III)^−^, and Sn(IV)^−^ perovskite materials, have been fabricated.^14−22^ In this context, low-dimensional all-inorganic copper halides have been extensively studied because of their multiple structural variations, high PLQY, and large Stokes shift. More importantly, the rapid development of all-inorganic copper halides for X-ray detection and imaging has led to extended research into their organic copper halide counterparts, which exhibit significantly improved environmental stability and distinct optoelectronic properties, such as more localized excited electronic states and stronger exciton–phonon coupling.^23−26^

Nevertheless, due to the strong light scattering at grain boundaries of copper halide/polymer composite scintillation screens, they consistently exhibit a lower spatial imaging resolution compared with other materials at the same light yield (Scheme 1a). Therefore, strategies that can improve the spatial resolution of X-ray scintillation screens to suppress light scattering are highly desirable. Recently, it has been reported that scintillator arrays embedded in anodized aluminum oxide (AAO) templates possess optical waveguide structures that confine light in high refractive index pores and reduce optical cross-talk, thus enabling excellent scintillation and X-ray imaging performance.^27,28^ Compared with AAO templates, silicon templates with a larger area and tunable micropores can effectively obviate solution-processed pixelated scintillator fabrication methods. Furthermore, although light management engineering has proven to be effective in improving imaging resolution, little attention has been paid to the effect of template structure on imaging resolution. We conclude that thinner silicon templates with smaller pore diameters can significantly reduce light scattering and spreading, improving the resolution of X-ray imaging screens (Scheme 1b,c).

**Scheme 1:**
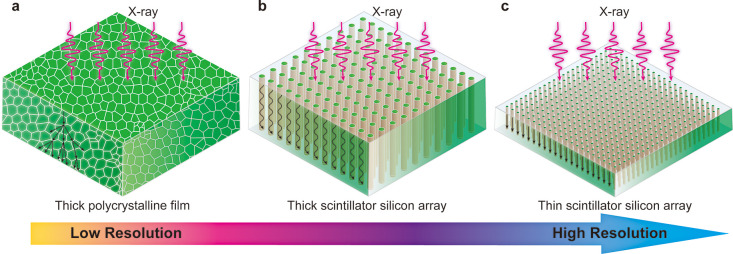
Pixelated Screens Confine Scintillating Light in Silicon Pores through Total Internal Reflection and Enhance Imaging Resolution (a) Low X-ray imaging resolution due to the light scattering of particles in the thick polycrystalline scintillator screen, (b) the optical waveguide effect of the thick silicon array scintillator screen, which reduces light scattering and improves the spatial resolution, and (c) a thin silicon array scintillator screen with lower light scattering that enables a higher spatial resolution.

Herein, we present a zero-dimensional organic copper iodide, CNCI, with a high X-ray absorption cross-section and near-unity PLQY as a new high-performance X-ray imaging scintillator. The CNCI scintillator shows high X-ray sensitivity, including a high light yield of 87,500 photons/MeV and an ultralow detection limit of 59.4 nGy/s. More importantly, a pixelated CNCI-silicon array scintillation screen was successfully prepared by a facile solution synthesis route using a silicon template. Benefiting from the highly reduced light scattering and light confinement of the arrayed scintillator, the pixelated scintillator array exhibits a remarkably high spatial resolution of 24.8 lp/mm, demonstrating the distinct advantages of organic copper iodide materials in high-performance X-ray imaging applications.

Crystals of (18-crown-6)_2_Na_2_(H_2_O)_3_Cu_4_I_6_ (CNCI) were synthesized according to the ball milling method.^25^ CNCI crystallizes in a monoclinic space group (Figure 1a), with isolated [Cu_4_I_6_]^2–^ tetrahedrons surrounded by (18-crown-6)_2_Na_2_(H_2_O)_3_^2+^ cations. The structure of CNCI crystals and powder samples was well confirmed. The powder X-ray diffraction (XRD) pattern matches well with the standard reference (Figure S1), and EDS elemental mapping shows that Na, Cu, and I were homogeneously distributed in a crystal sample (Figures 1e–h and S2–S3). As shown in Figures 1b and S4, CNCI powder exhibited an absorption spectrum from 375 to 500 nm and a broad emission band from 450 to 750 nm centered at 536 nm, with a full width at half-maximum (FWHM) of 110 nm. It is worth noting that CNCI exhibits asymmetric emission spectra that correspond to the double emission of two different energy states: emission induced by metal–ligand charge transfer or halide-ligand charge transfer (MLCT/HLCT) at 536 nm and emission induced by a cluster center (CC) at 700 nm (Figure 1c). In addition, the PLQY (Figure S5) of the greenish-yellow emission was measured to be about 94%, which might originate from individual [Cu_4_I_6_]^−2^ quantum dots exhibiting a strong quantum confinement effect due to the 0D electronic nature of CNCI.^25,29,30^ The PL lifetime of the as-prepared CNCI crystals at 535 nm was then recorded by the time-correlated single-photon counting (TCSPC) method; the brief lifetime of 1.98 μs is shorter than that of most reported low-dimensional organic metal halides (Figure 1d).^31−33^ Due to their negligible self-absorption, ultrabroad emission spectrum, nearly 100% PLQY, and short luminescence lifetime, the CNCI crystals position them as a promising scintillator material.

**Figure 1:**
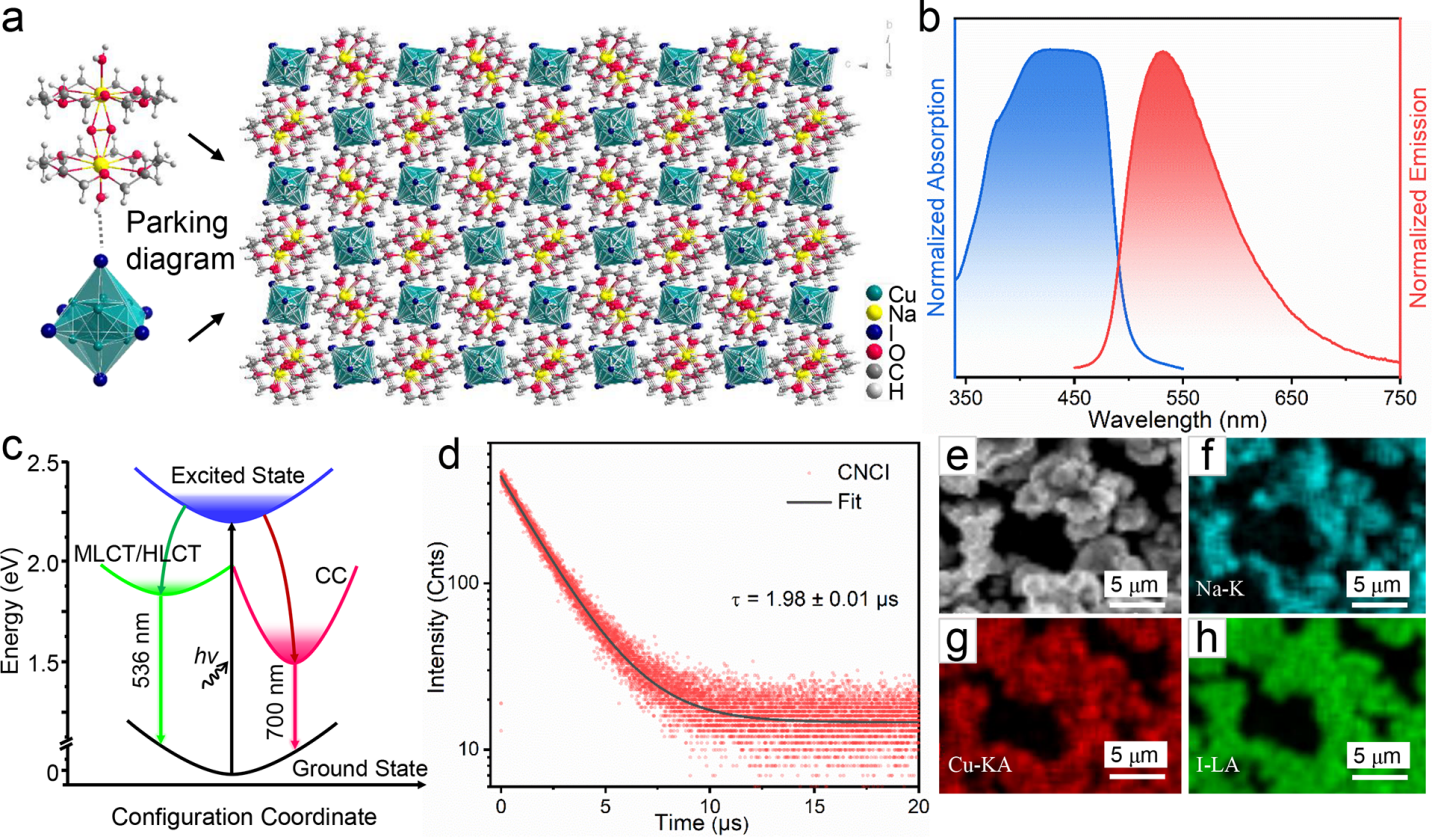
(a) Structure of 0D (18-crown-6)_2_Na_2_(H_2_O)_3_Cu_4_I_6_ (blue octahedrons: [Cu_4_I_6_]^2–^ octahedrons). (b) Absorption and photoluminescence (λ_ex_ = 420 nm) spectra of CNCI powder. (c) Schematic diagram of photophysical processes. (d) PL decay curve of CNCI under 450 nm excitation and monitoring wavelength of 535 nm. (e–h) Energy dispersive X-ray spectroscopy (EDS) elemental mapping of CNCI powders with the scale bar of 5 μm.

With respect to scintillation performance, the absorption spectrum of CNCI was calculated and found to be comparable to that of commercial scintillators (LYSO) over a broad range of X-ray detection regions (4–60 keV) (Figure 2a).^34^ CNCI-polysulfone (CNCI-PSF) composite films were first engineered by encapsulating different weight percentages of CNCI particles into a polysulfone matrix. As shown in Figure 2b, the radioluminescence (RL) spectra of the CNCI-PSF composite films showed peaks similar to those observed in the corresponding PL spectra, indicating the same luminescence mechanism under UV and X-ray irradiation. Moreover, the RL intensity gradually increased as the doping ratio of CNCI in PSF increased from 50 to 70 wt.% due to more efficient X-ray absorption at higher concentrations. According to Figure S6, the attenuation efficiencies of 500 μm LYSO crystal and CNCI-PSF film are 90% and 72%, respectively. By normalizing the absorption of X-rays by the two scintillators, it can be ensured that the CNCI absorbs the same X-rays photons as the commercial LYSO film. The light yield (∼109,000 photons/MeV) of the 70 wt.% CNCI-PSF film was calculated by integrating the X-ray-induced RL spectra and comparing the results with those obtained for the references LYSO:Ce (33,000 photons per MeV) and Bi_4_Ge_3_O_12_ (BGO) (10,000 photons per MeV) (Figure 2c). The light yield of CNCI significantly outperforms that of previously reported CsPbBr_3_ nanocrystals (∼21,000 photons/MeV),^9^ Rb_2_CuBr_3_ (∼91,000 photons/MeV),^14^ and (C_38_H_34_P_2_)MnBr_4_ (∼80,000 photons/MeV)^16^ and is even higher than that of some commercial scintillators (∼CsI:Tl: 66,000 photons/MeV; GOS: ∼60,000 photons/MeV),^35,36^ demonstrating the material’s high potential in medical radiography and security screening.

**Figure 2:**
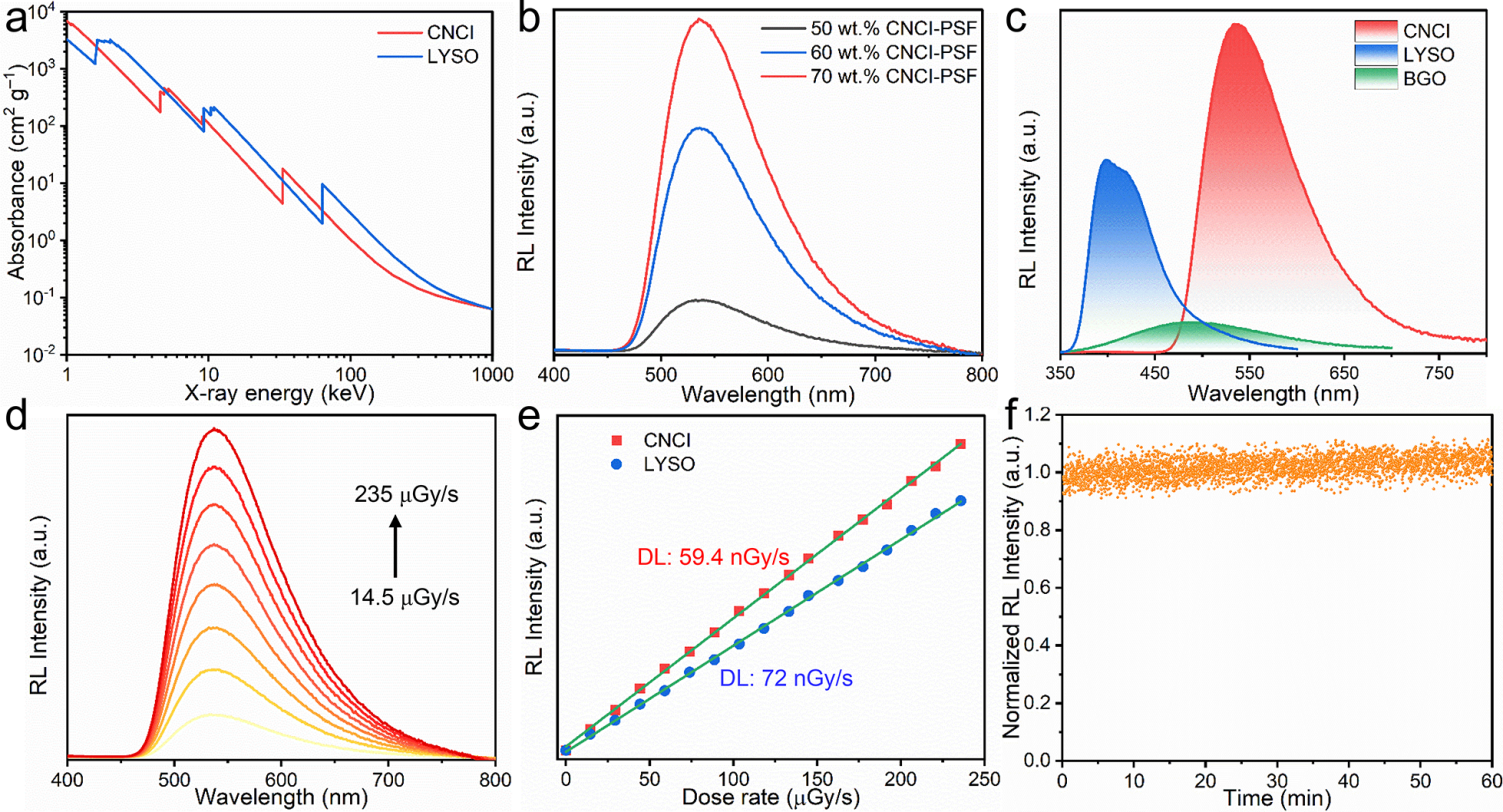
(a) Absorption coefficients of CNCI and LYSO under high-energy radiation. (b) Dependence of the radioluminescence intensity of CNCI-PSF films on the concentration of CNCI. (c) Radiation luminance spectra of CNCI films, LYSO:Ce, and BGO single crystals under the same X-ray illumination. (d) Radioluminescence spectra of 70 wt.% CNCI-PSF film under X-ray radiation at different dose rates. (e) Radioluminescence intensity of 70 wt.% CNCI-PSF film and LYSO:Ce as a linear function of dose rate. (f) Radioluminescence stability under continuous X-ray illumination at a dose of 2.4 mGy/s.

The RL intensity of the 70 wt.% CNCI-PSF film scaled linearly with the X-ray dose rate from 14.5 μGy/s to 235 μGy/s (Figure 2d). A low detection limit of 59.4 nGy/s was calculated from the slope of the fitting line in Figure 2e and Figure S7 at a signal-to-noise ratio of 3, which is lower than that obtained for the LYSO scintillator (72 nGy/s) and nearly 92 times lower than the dose rate required for a standard medical X-ray diagnosis (5.5 μGy/s). Moreover, the RL intensity of the 70 wt.% CNCI-PSF film remained at ∼100% of the initial value over 1 h of continuous X-ray irradiation at a dose rate of 2.4 mGy/s (total dose, 8.6 Gy) (Figure 2f), demonstrating the high structural and radiation stability of the CNCI scintillator during extended radiation exposure.

To further validate the potential of CNCI as a scintillation material for practical X-ray imaging, we encapsulated CNCI in polysulfone polymer film and silicon membranes. X-ray imaging tests were then performed on a home-built imaging system consisting of an X-ray source, an imaging object, a reflector, and a camera. As shown in Figures 3f and S8, a distinguishable line spacing between 14.3 and 16.6 lp/mm was obtained using 70 wt.% CNCI-PSF scintillation films measuring 100 μm in thickness. A spatial resolution of 16.3 lp/mm was calculated at a modulation transfer function (MTF) value of 20%, consistent with the resolution limit observed from the standard line-pair card (Figure 3j).

**Figure 3:**
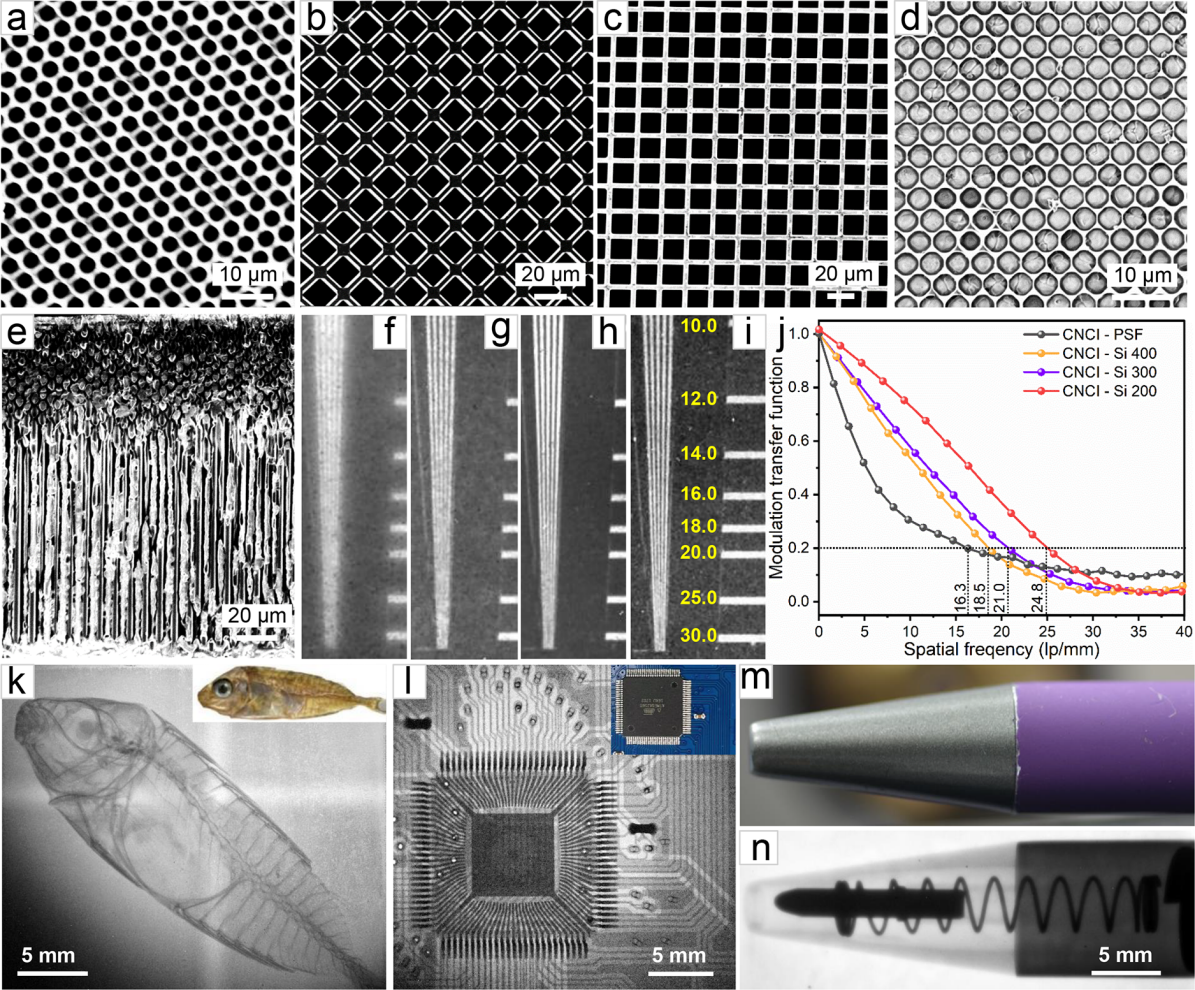
(a–c) Top-view SEM images of silicon 200, 300, and 400 templates, respectively. (d) Top-view and (e) cross-sectional SEM image of as-prepared CNCI-silicon 200 array scintillator. Two-dimensional X-ray images of a test-pattern plate based on (f) CNCI-PSF film, (g) CNCI-Si 400, (h) CNCI-Si 300, and (i) CNCI-Si 200 films. (j) MTF of CNCI-PSF and CNCI-Si array films measured by the slanted-edge method. Photographs (insets) and X-ray images of (k) fish, (l) circuit board, and (m, n) pen.

More importantly, the X-ray imaging resolution of CNCI was further improved by confining the material in silicon membranes with controllable pore size and structure due to guiding the scintillating lights in high refractive index pores. The CNCI scintillator crystals were well encapsulated in the pores of silicon membranes of different thicknesses by using the impregnation method (Figures 3a–c and S9). Top and cross-sectional SEM images (Figures 3d,e, and S10–S12) show that the pores of the silicon membranes were completely filled with CNCI crystals, and one-dimensional scintillator rod arrays with different diameters (17 μm, 17 μm, and 2.8 μm for CNCI-Si 400, CNCI-Si 300, and CNCI-Si 200) were well formed. Interestingly, the X-ray imaging resolution was highly increased from 16.3 lp/mm for the CNCI-PSF films to 18.5 lp/mm for the CNCI-Si 400 screen (Figures 3g,j, and S13). Moreover, the imaging resolution of the pixelated CNCI-silicon array scintillation screen could be further improved by adjusting the thickness and pore size of the Si template. As shown in Figures 3h and S14, the spatial imaging resolution of CNCI-Si 300 films was improved to 20.3 lp/mm when the thickness of the silicon template was reduced to 300 μm. Further decreasing the thickness and hole diameter of the silicon templates led to a remarkably increased spatial resolution of 24.8 lp/mm for CNCI-Si 200 films with a thickness of 200 μm and a hole diameter of 2.8 μm (Figures 3i and S15). We found that the light output of the CNCI-Si scintillator screens decreases as the silicon template becomes thinner due to the reduced X-ray absorption by the CNCI-Si screens (Table S1). In addition, the light output of CNCI-Si scintillator screens is lower than that of pure CNCI films. This is because the silicon template will also absorb the light emitted by the CNCI scintillator under X-ray radiation. Therefore, we conclude that the thickness and hole diameter of the silicon template can significantly affect the light propagation process of the scintillators at the microscale domain. By decreasing the thickness and pore diameter of the silicon template, more efficient light propagation will occur. This is the main reason for the increased spatial resolution observed on CNCI-Si 200 scintillators screens. Based on the ultrahigh resolution of the pixelated CNCI-silicon array scintillation screen, we performed a series of imaging experiments to demonstrate their high practical application value in radiography. The skeleton of a small fish, the internal circuitry of a microchip, and the spring inside a ballpoint pen were clearly distinguished by a well-defined boundary under X-ray irradiation by using the CNCI-Si 200 scintillation screen (Figure 3k). These results demonstrate the great potential of the CNCI-Si array scintillation films in biological imaging and nondestructive detection (Figure 3l–n).

In summary, we successfully developed novel organometallic halide X-ray scintillators based on (18-crown-6)_2_Na_2_(H_2_O)_3_Cu_4_I_6_ with negligible self-absorption and near-unity green-light emission. The CNCI scintillators exhibited desirable X-ray sensitivity, including an ultrahigh light yield of ∼109,000 photons/MeV and an ultralow detection limit of 59.4 nGy/s, which is approximately 92 times lower than the dosage for a standard medical diagnosis. In addition, a facile solution-embedding strategy was proposed to fabricate an array scintillation screen based on silicon membranes. We found that the thickness and pore diameter of the silicon template can significantly influence the spatial resolution of the CNCI-Si scintillator array screen. As a consequence, a CNCI-Si 200 scintillator array demonstrated excellent light propagation efficiency in micron-size pores and achieved the highest spatial imaging resolution of up to 24.8 lp/mm. Our study is a pioneering effort in the structure-to-performance correlation of scintillator arrays designed for use in X-ray imaging and may stimulate the exploration of low-cost, high-performance, eco-friendly metal halide materials as radiation scintillators.

## Supporting Information Available

Experimental methods; additional X-ray imaging data; composition and structural characterization by XRD, UV–vis, and SEM (PDF)
