# Supplementary material for: Copper Organometallic Iodide Arrays for Efficient X-ray Imaging Scintillators
Source: ACS Cent Sci. 2023 Mar 10;9(4):668–74. doi: 10.1021/acscentsci.2c01495 (PMC10141593; doi:10.1021/acscentsci.2c01495)
Supplement: Supplementary file 1 — oc2c01495_si_001.pdf [file oc2c01495_si_001.pdf]

Supporting Information

Copper Organometallic Iodide Arrays for Efficient X-ray Imaging Scintillators

Hong Wang, [†,‡] *Jian-Xin Wang,* [†] *Xin Song,* [‡] *Tengyue He,* [†,‡] *Yang Zhou,* [†,‡] *Osama Shekhah,* [†] *Luis Gutiérrez-Arzaluz,* [†,‡] *Mehmet Bayindir,* [§] *Mohamed Eddaoudi,* [†] *Osman M. Bakr,* [‡] *and Omar F. Mohammed*[*,†,‡]

[†] Advanced Membranes and Porous Materials Center, Division of Physical Science and Engineering, King Abdullah University of Science and Technology, Thuwal 23955-6900, Kingdom of Saudi Arabia

[‡] KAUST Catalysis Center, Division of Physical Sciences and Engineering, King Abdullah University of Science and Technology, Thuwal 23955-6900, Kingdom of Saudi Arabia

[§] Center for Hybrid Nanostructures, University of Hamburg, 22761 Hamburg, Germany

[*]Correspondence and requests for materials should be addressed to Omar F. Mohammed. (email: omar.abdelsaboor@kaust.edu.sa).

Chemicals and Materials. Copper (I) iodide (CuI, 99.999%), sodium iodide (NaI, 99%), 18-crown-6 ($C_{12}H_{24}O_6$, 99%), and Poly sulfone (average Mw ~350,000 by GPC) were purchased from Sigma Aldrich. H_3PO_2 (50 wt. %, in water) was received from Alfa Aesar. Chloroform (HPLC) was received from VWR Chemicals. Silicon templates were received from Smart MEMBRANES. These chemicals and reagents were used as received without further purification.

Synthesis of (18-crown-6)$_2$Na$_2$(H$_2$O)$_3$Cu$_4$I$_6$ (CNCI) Powder. CuI (2.5 mmol), NaI (2.5 mmol), H_3PO_2 (0.05 mL), and 18-crown-6 (5.0 mmol) were mixed in the mortar. The reaction mixture was ground for 10 min at room temperature to form the light green paste. The paste was dried naturally under the air.

Fabrication of CNCI-Polysulfone Composite Films. A calculated amount of CNCI powder was added in 2 mL of chloroform solvent and sonicated for ca. 2 min. Then, 200 mg Polysulfone (PSF) was added to the solution, followed by sonication for 2 min and shaking for 3 h to ensure that the powder and the polymer were well mixed. The viscous solution was carefully coated on the quartz plates (or in a 20 mL glass vial) and then covered with a beaker to allow the solvent to evaporate slowly in order to obtain the film with a uniform surface and good morphology.

Fabrication of CNCI-Si Pixelated Scintillating Screens. The CNCI powder was added to DMF solution and sonicated for about 5 min to dissolve completely. Then, a clean silicon template was added to the DMF solution and sonicated for 10 min to ensure that the solution was fully immersed in the silicon pores. The silicon template was removed from the solution and dried on a 100°C hot plate.

Characterizations of Samples. X-ray diffraction (XRD) patterns were recorded at room temperature using a Bruker D8 ADVANCE diffractometer with Cu Kα radiation (λ = 1.5406 Å). Scanning electron microscopy (SEM; Zeiss Auriga) with an energy-dispersive X-ray analyzer was used to analyze the morphologies and the chemical compositions of powder. Absorption spectra of the samples were measured using a UV-Vis spectrometer (PerkinElmer Lambda 950) equipped with an integrated sphere. The photoluminescence (PL) spectra were taken using a Horiba Fluoromax-4 spectrofluorometer with a photomultiplier (PMT-928). Photoluminescence quantum yield (PLQY) of the samples was measured using FS5 fluorescence spectrometer (Edinburgh Instruments). The excitation wavelength was set at 420 nm. Time-resolved photoluminescence was obtained through the TCSPC technique in a setup based on a modified microscope (Olympus IX71). The sample was excited at 450 nm with a pulsed diode laser (70 ps, HORIBA, Delta Diode) focused through a 10×, 0.4 NA microscope objective (Olympus); it was also used to collect the PL. Long-pass 493 nm and band-pass 535 nm filters (Newport) were used to reject scattered laser light and select the emission detection range. The filtered PL signal was focused on an avalanche photodiode (PDM series, MicroPhoton Devices). The histograms obtained were fitted with the SymphoTime64 software (PicoQuant) using the Levenberg–Marquardt iteration algorithm. The overall system's time resolution was better than 150 ps.

Radioluminescence (RL) Studies. The RL spectra were taken by a spectrometer (Horiba Fluoromax-4 spectrofluorometer) equipped with an X-ray tube (Tungsten target, Moxtek). The detection slit was set at 1 nm, and the X-ray outlet was set to 1 cm away from sample for all spectral measurements. The X-ray dose rate was controlled in a range from 3.43 μGy/s to 247

μGy/s by adjusting the input current and voltage values. All tests were carried out in a radiation-tight environment of lead-plate shielding.

Calculation of X-ray Detection Limit. The linear relationship between the RL intensity of the corresponding samples and the X-ray dose was obtained. The noise data was obtained in the absence of sample. The noise intensity value was statistically analyzed and fitted by the Gaussian function, whereby the FWHM was regarded as the average value of the noise. The detection limit in dose rate was derived from the slope of the fitting line, with a signal-to-noise ratio of 3.

Calculation of X-ray Light Yield. Commercial LYSO:Ce scintillator was used as a reference to estimate the light yield of the film samples. The films were fabricated to keep the same size (2 × 2 cm^2) and thickness (500 μm) as the LYSO:Ce reference sample, and RL curves were obtained using spectrometer under an identical configuration. Typically, the light yield of 70 wt. % CNCI-PSF film was estimated around 109,000 photons/MeV. BGO scintillator was used as another reference to cross-check the accuracy of this measurement method. The light yield is calculated by taking into the account the wavelength dependent detector efficiency (Hamamatsu, R928 PMT), X-ray absorption efficiencies of the LYSO:Ce and CNCI-PSF composite:

$$\mathrm{LY}_S = \mathrm{LY}_R \frac{\int \mathrm{I}_S(\lambda)\mathrm{d}\lambda}{\int \mathrm{I}_R(\lambda)\mathrm{d}\lambda} \frac{\int \mathrm{I}_R(\lambda)\mathrm{S}(\lambda)\mathrm{d}\lambda}{\int \mathrm{I}_S(\lambda)\mathrm{S}(\lambda)\mathrm{d}\lambda} \frac{\mathrm{XAE}_R}{\mathrm{XAE}_S}$$

Where LY_S and LY_R are sample and reference light yields, $\mathrm{I}_S(\lambda)$ and $\mathrm{I}_R(\lambda)$ are wavelength dependent radioluminescence intensities of sample and reference, $\mathrm{S}(\lambda)$ is wavelength dependent detector efficiency, XAE_S and XAE_R are X-ray attenuation efficiencies of sample and reference.

X-ray Image Collection and Process. A commercial camera (D7100, Nikon) was used to take images for X-ray imaging screen. The ISO, aperture, and shutter were set at 640, F3.5, and 10 for samples excited by X-ray. The voltage/current of the X-ray tube was set to 50 kV/200 μA (dose rate ≈ 5.2 mGy/s. The original X-ray images were further processed using the software Image J to remove the background of screen.

Calculation of X-ray Imaging Spatial Resolution. X-ray Imaging spatial resolution was calculated by Modulation transfer function (MTF) measurements. The MTF was calculated by the slanted edge method. Sharp edge X-ray imaging was carried out on the standard line-pair lead template with a thickness of ~ 0.5 mm. MTF operation on images through software ImageJ: Then the edge spread function (ESF) was derived from the edge image, and the line spread function (LSF) was derived from the derivation. Finally, the Fourier transform of LSF defined MTF. The summary was shown in the following formula:[1]

$$MTF(v) = F(LSF(x)) = F(\frac{dESF(x)}{dx})$$

Where ν was the spatial frequency. X is the position of pixels.

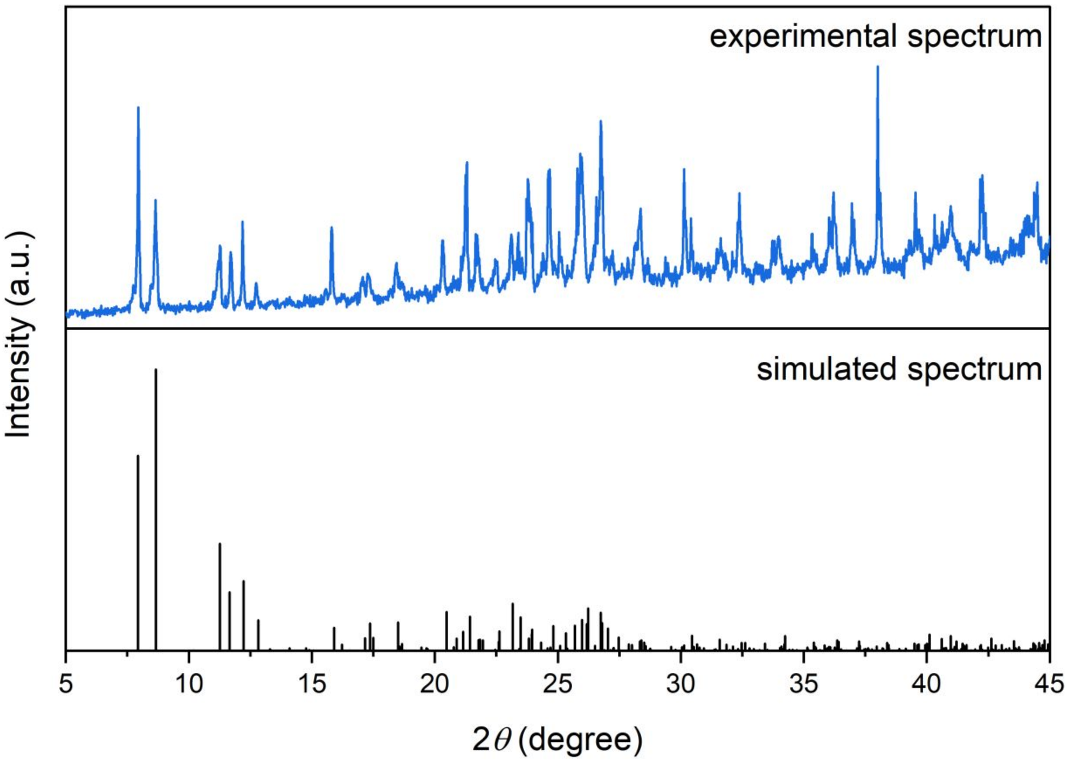

Figure S1. XRD patterns of the CNCI powders.

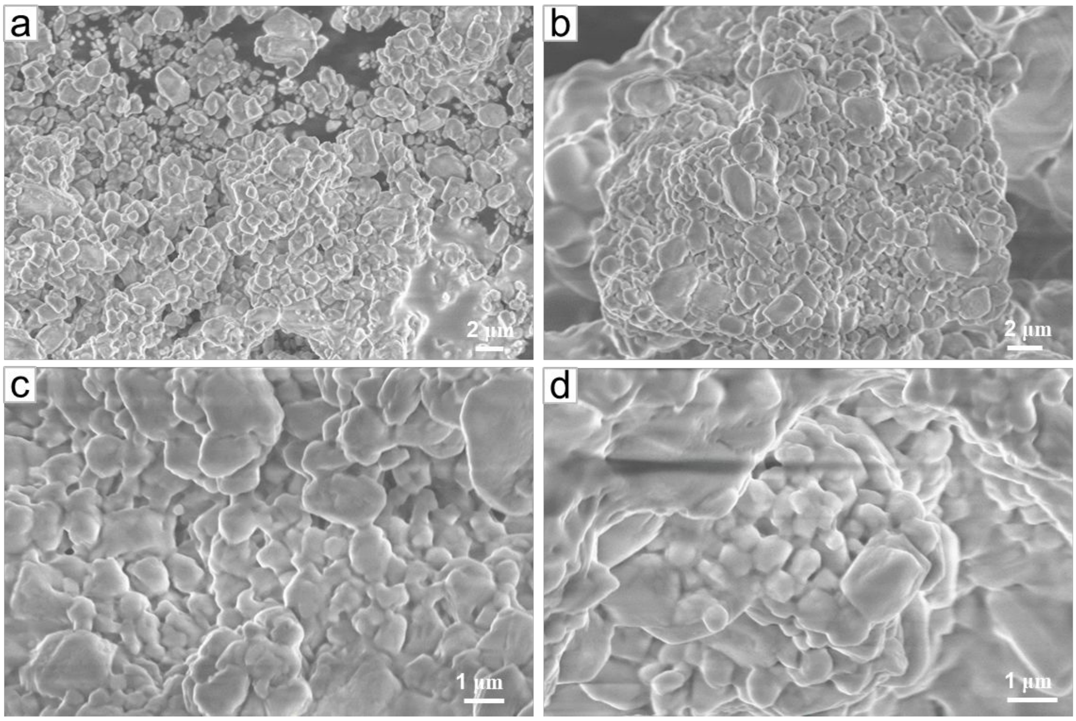

Figure S2. Scanning Electron Microscope (SEM) images of the as-synthesized CNCI powder with the scale bar of 2 μm (a-b) and 1 μm (c-d).

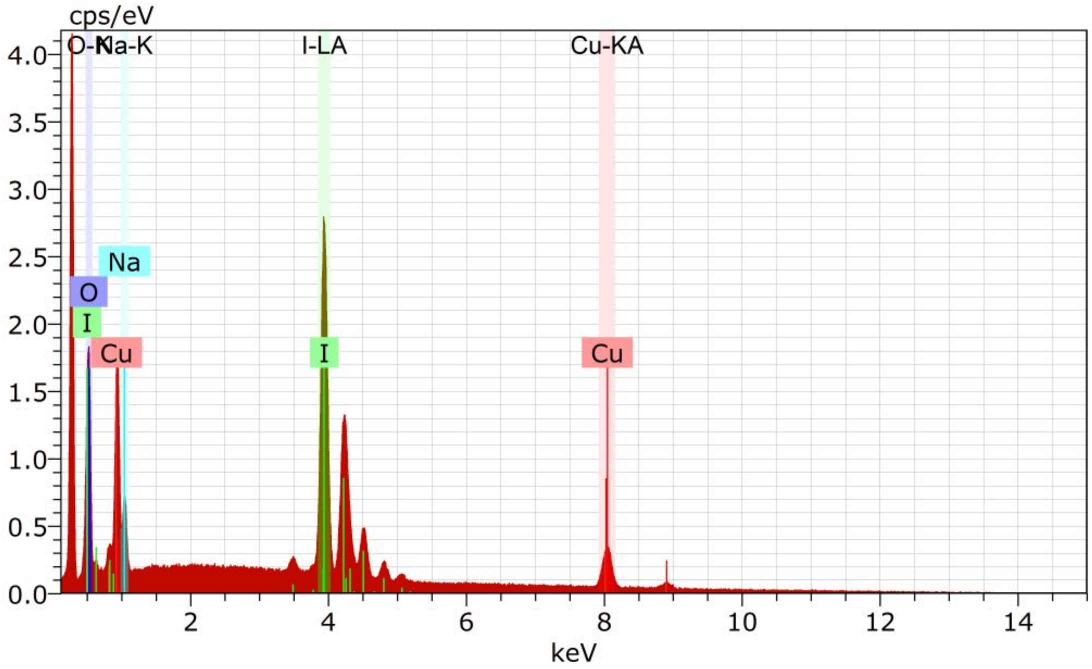

Figure S3. EDS spectra of the CNCI powders.

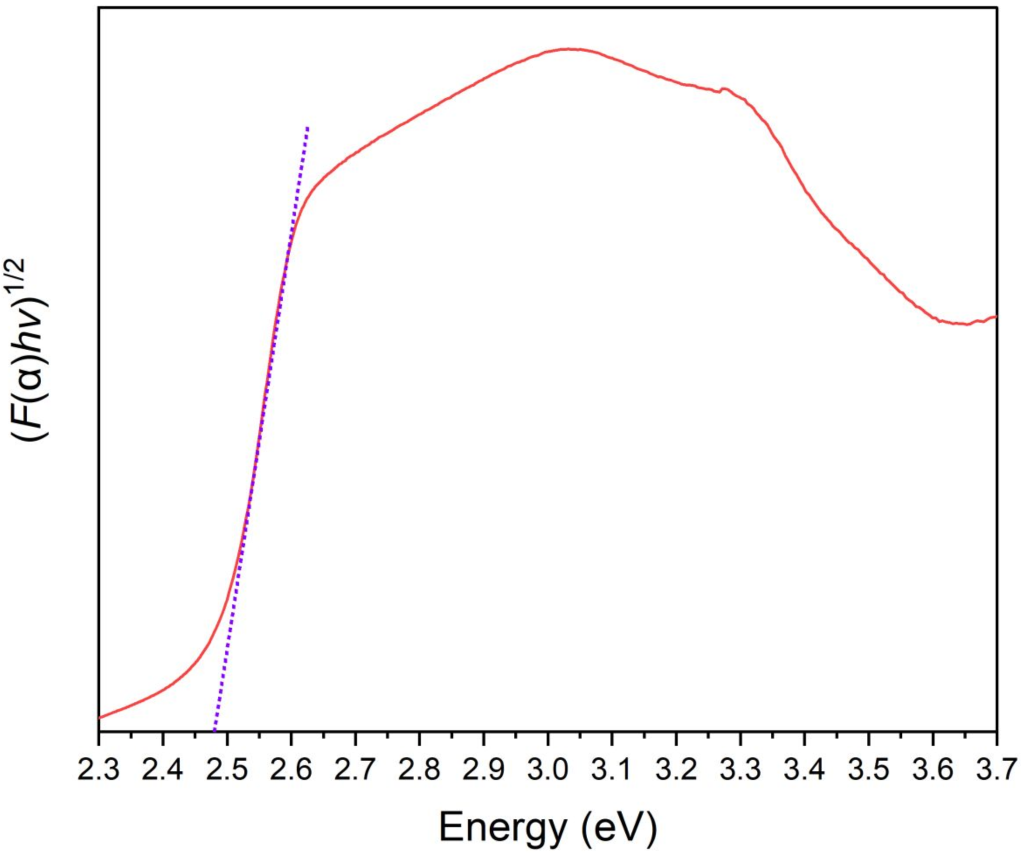

Figure S4. Kubelka-Munk equation applied to the absorbance spectrum of the CNCI powder.

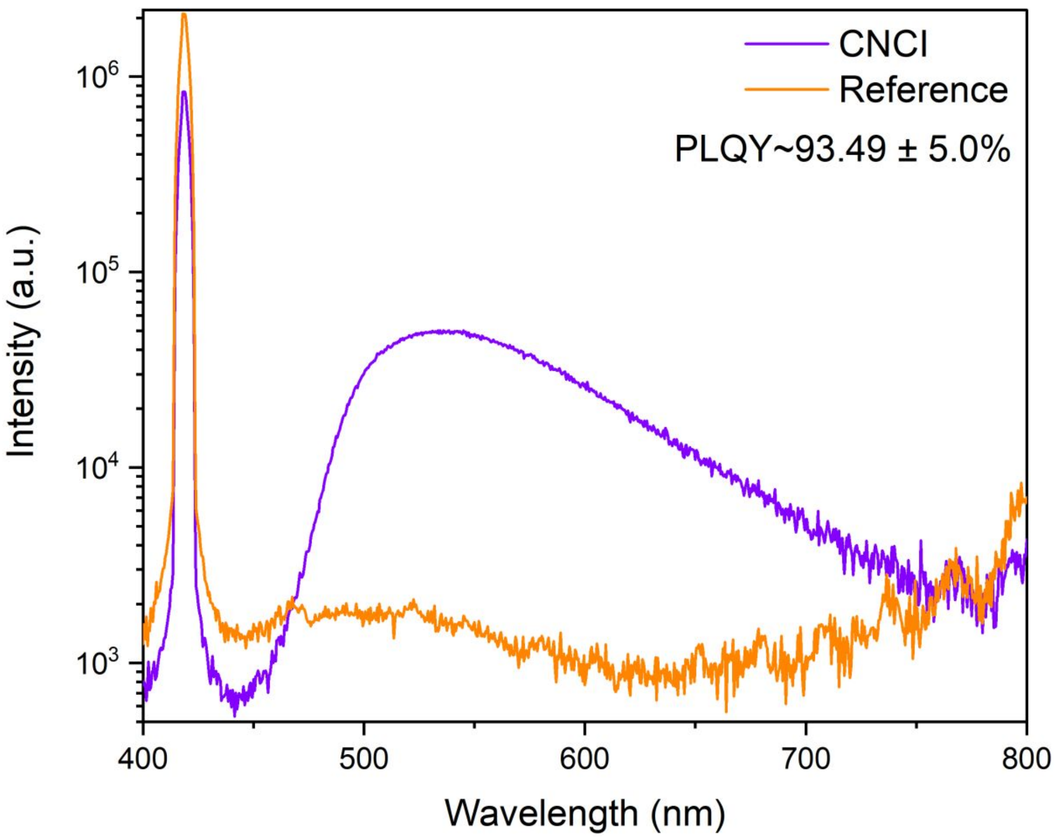

Figure S5. The PLQY spectra of CNCI powder. The reference curves were measured by placing a blank sample holder in the integrating sphere.

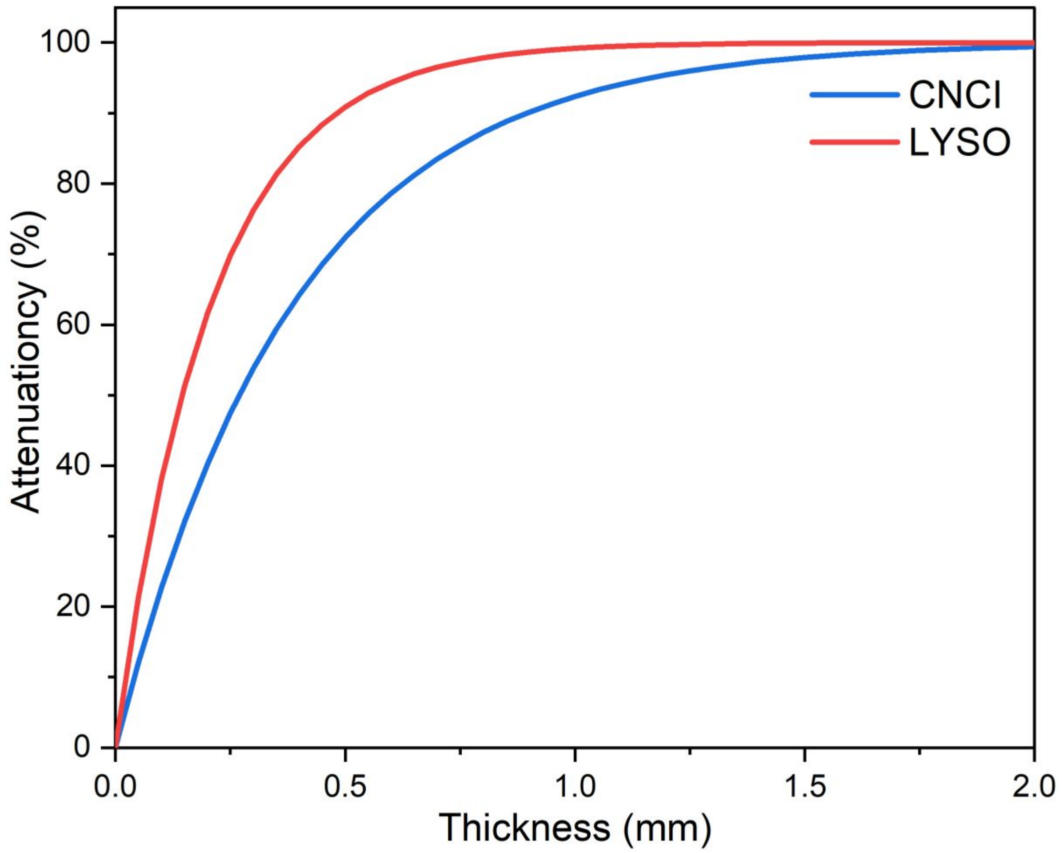

Figure S6. Attenuation efficiency of CNCI and LYSO for 40 keV X-ray photons versus thickness.

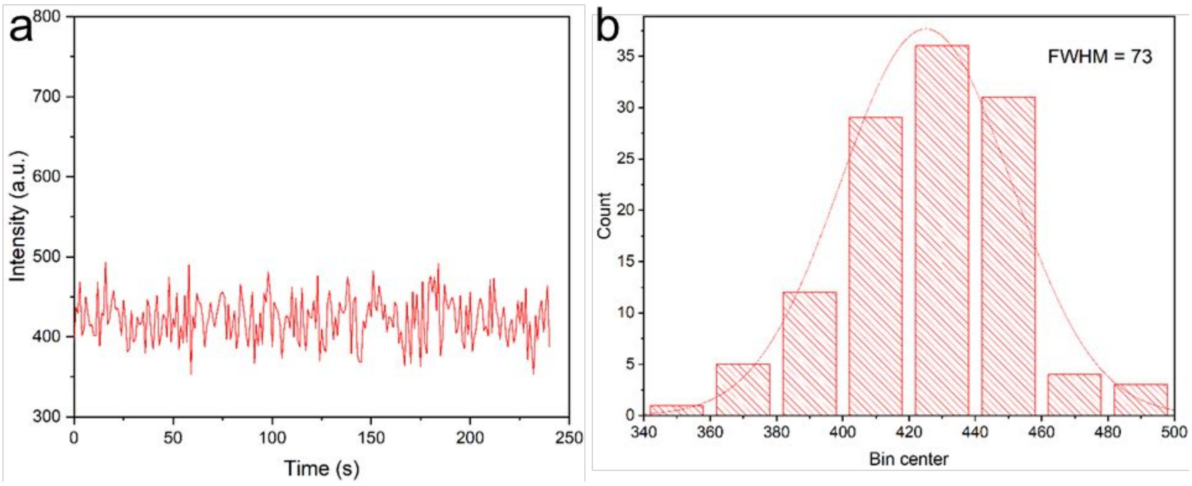

Figure S7. a) Background signal of the RL spectra. b) Background signal fitted with Gaussian function (Full width at half maximum: FWHM = 73).

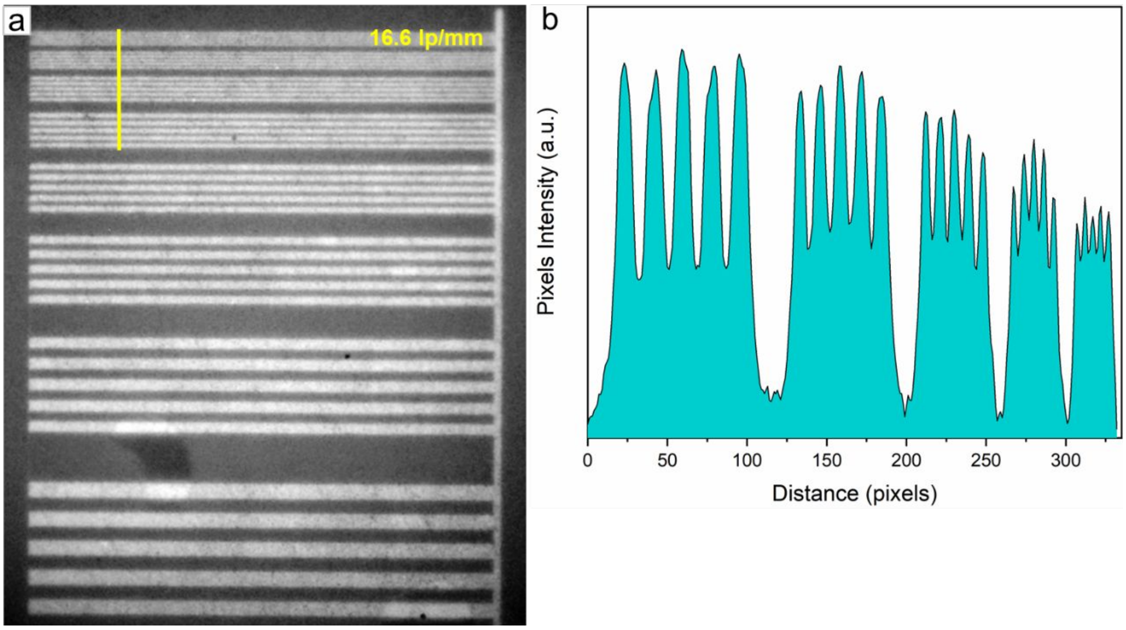

Figure S8. a) Spatial resolution of the 70 wt. % CNCI-PSF film was determined by a standard line-pair card (lp/mm). b) Spatial resolution measurement by the fitting of intensity spread profile.

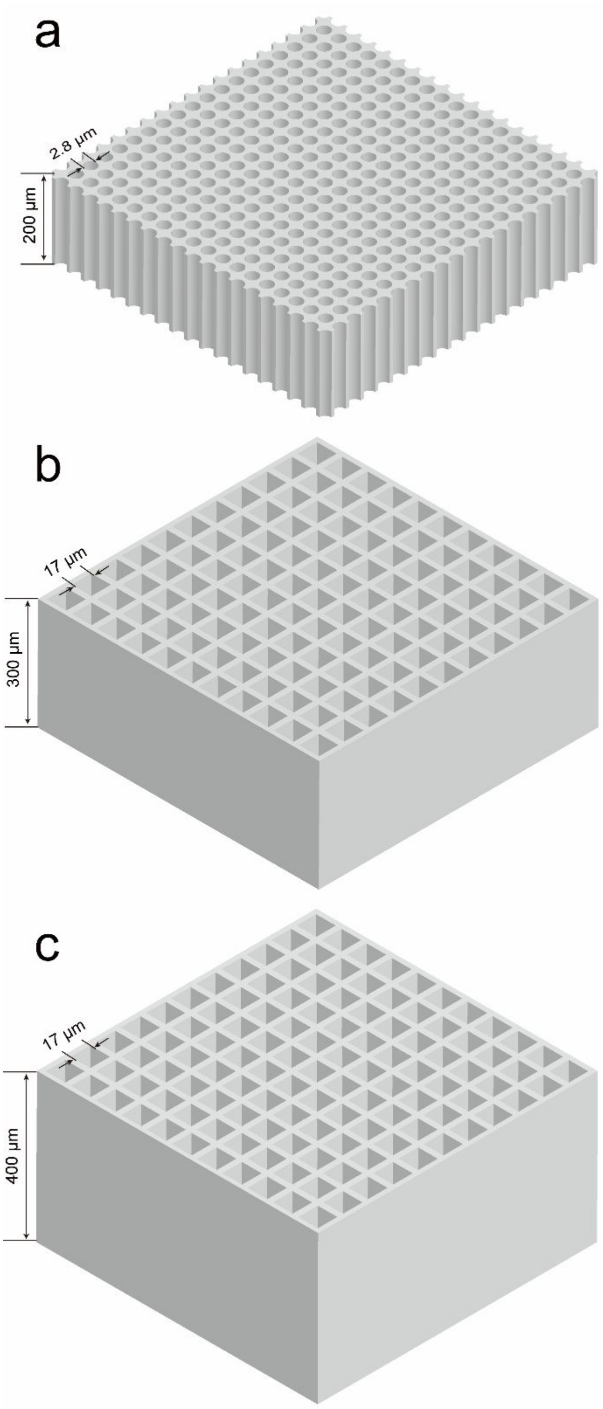

Figure S9. Schematic diagram of the structure and size of the (a) Si 200, (b) Si 300, and (c) Si 400 templates.

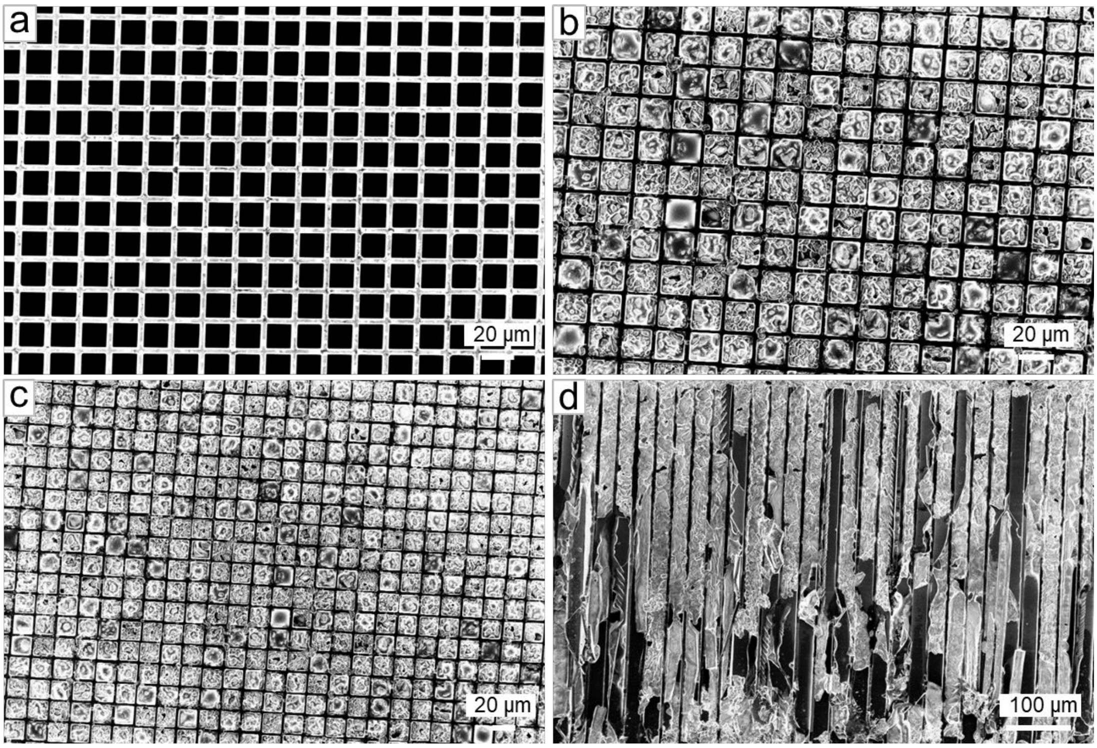

Figure S10. Surface SEM of Si 400 template, b) surface SEM of the CNCI-Si 400 arrays, c) surface SEM of the CNCI-Si 400 arrays, d) cross-sectional SEM image of CNCI-Si 400 arrays.

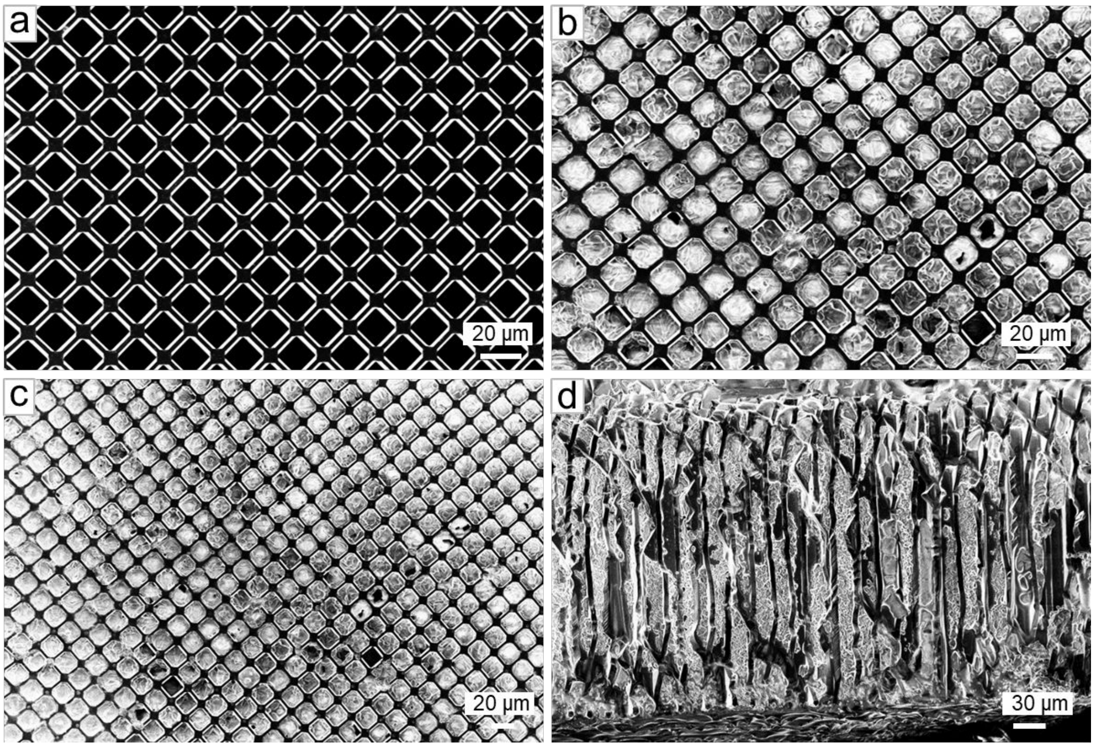

Figure S11. Surface SEM of Si 300 template, b) surface SEM of the CNCI-Si 300 arrays, c) surface SEM of the CNCI-Si 300 arrays, d) cross-sectional SEM image of CNCI-Si 300 arrays.

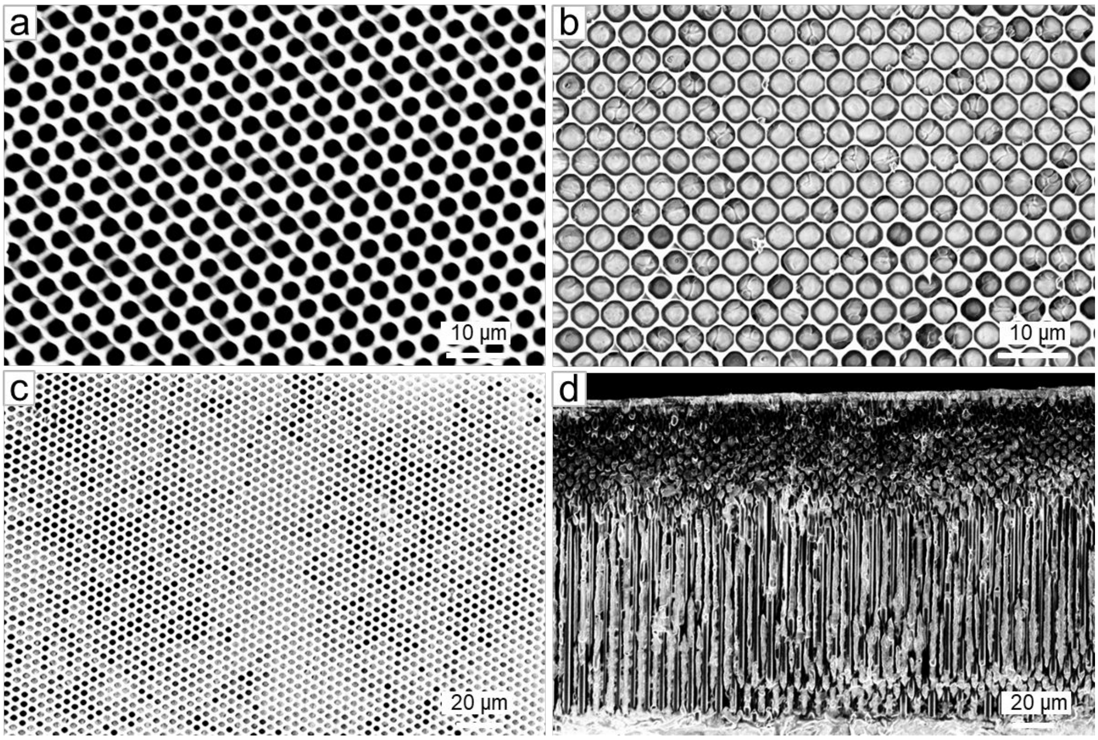

Figure S12. Surface SEM of Si 200 template, b) surface SEM of the CNCI-Si 200 arrays, c) surface SEM of the CNCI-Si 200 arrays, d) cross-sectional SEM image of CNCI-Si 200 arrays.

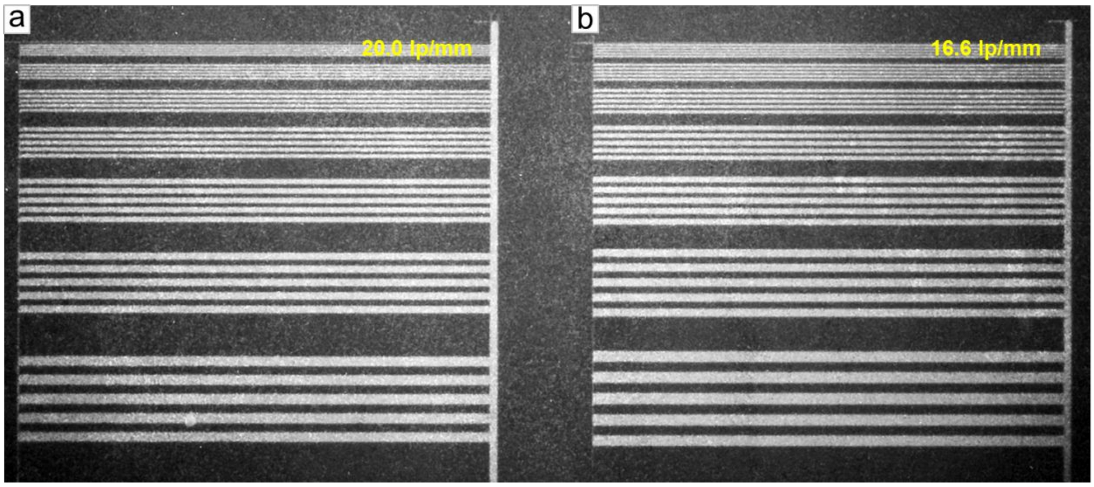

Figure S13. Spatial resolution of the CNCI-Si 400 arrays was determined by a standard line-pair card (lp/mm).

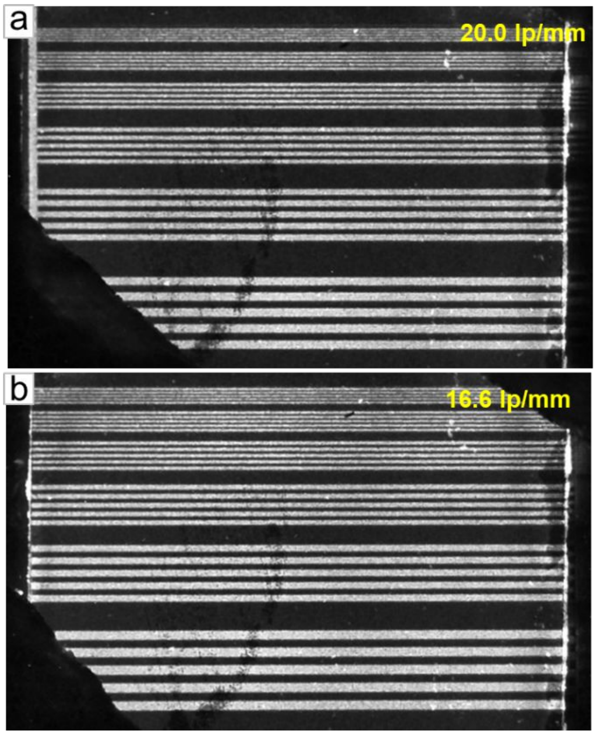

Figure S14. Spatial resolution of the CNCI-Si 300 arrays was determined by a standard line-pair card (lp/mm).

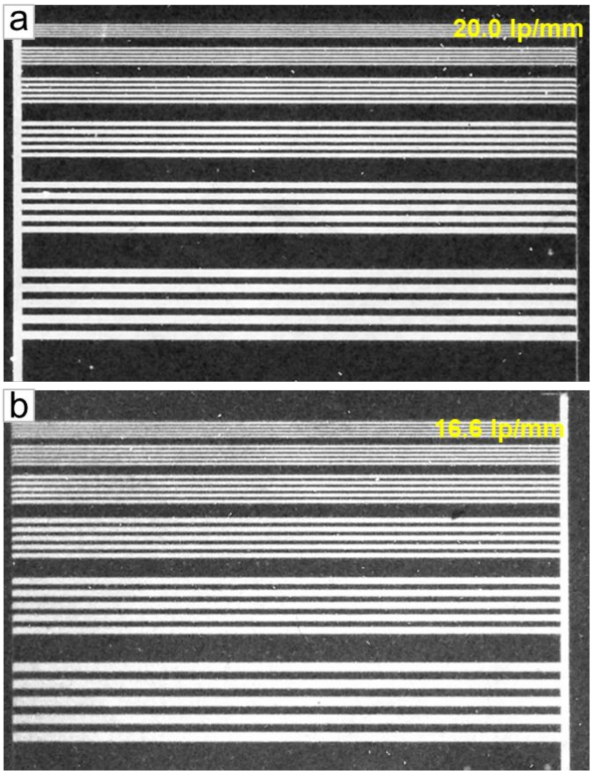

Figure S15. Spatial resolution of the CNCI-Si 200 arrays was determined by a standard line-pair card (lp/mm).

	CNCI-Si 200	CNCI-Si 300	CNCI-Si 400	CNCI-PSF unnormalized	CNCI-PSF normalized
Light output (photons/MeV)	~16 600	~34 100	~43 700	~87 500	~109 000

Table S1. The light output of different CNCI-Si and CNCI-PSF scintillators.

REFERENCES

1. Chen, W.; Zhou, M.; Liu, Y.; Yu, X.; Pi, C.; Yang, Z.; Zhang, H.; Liu, Z.; Wang, T.; Qiu, J.; Yu, S. F.; Yang, Y.; Xu, X. All-Inorganic Perovskite Polymer-Ceramics for Flexible and Refreshable X-Ray Imaging. *Adv. Funct. Mater.* **2021**, *32*, 2107424.
